# Tissue Harvesting Site and Culture Medium Affect Attachment, Growth, and Phenotype of *Ex Vivo* Expanded Oral Mucosal Epithelial Cells

**DOI:** 10.1038/s41598-017-00417-z

**Published:** 2017-04-06

**Authors:** Rakibul Islam, Jon Roger Eidet, Reza A. Badian, Marit Lippestad, Edward Messelt, May Griffith, Darlene A. Dartt, Tor Paaske Utheim

**Affiliations:** 1 Schepens Eye Research Institute/Massachusetts Eye and Ear, Harvard Medical School, Boston, MA USA; 2 Department of Oral Biology, Faculty of Dentistry, University of Oslo, Oslo, Norway; 3 Department of Medical Biochemistry, Oslo University Hospital, Oslo, Norway; 4 Department of Ophthalmology, Oslo University Hospital, Oslo, Norway; 5 Faculty of Visual Sciences, University College of Southeast Norway, Kongsberg, Norway; 6 Department of Ophthalmology, Innlandet Hospital Trust, Elverum, Norway; 7 Faculty of Medicine, University of Oslo, Oslo, Norway; 8 Integrative Regenerative Medicine (IGEN) Centre, Department of Clinical and Experimental Medicine, Linköping University, Linköping, Sweden

## Abstract

Transplantation of cultured oral mucosal epithelial cells (OMECs) is a promising treatment strategy for limbal stem cell deficiency. In order to improve the culture method, we investigated the effects of four culture media and tissue harvesting sites on explant attachment, growth, and phenotype of OMECs cultured from Sprague-Dawley rats. Neither choice of media or harvesting site impacted the ability of the explants to attach to the culture well. Dulbecco’s modified Eagle’s medium/Ham’s F12 (DMEM) and Roswell Park Memorial Institute 1640 medium (RPMI) supported the largest cellular outgrowth. Fold outgrowth was superior from LL explants compared to explants from the buccal mucosa (BM), HP, and transition zone of the lower lip (TZ) after six-day culture. Putative stem cell markers were detected in cultures grown in DMEM and RPMI. In DMEM, cells from TZ showed higher colony-forming efficiency than LL, BM, and HP. In contrast to RPMI, DMEM both expressed the putative stem cell marker Bmi-1 and yielded cell colonies. Our data suggest that OMECs from LL and TZ cultured in DMEM give rise to undifferentiated cells with high growth capacity, and hence are the most promising for treatment of limbal stem cell deficiency.

## Introduction

The integrity of the outermost layer of the cornea, the epithelium, is dependent on stem cells located in the corneal periphery, the limbus. These stem cells can be damaged by a number of diseases, but also external factors, such as those causing chemical and thermal burns. In limbal stem cell deficiency (LSCD), the cornea can become opaque and painful. Since 1997, LSCD has been treated successfully by transplanting cultured limbal epithelial stem cells from donors^1–3^. In bilateral LSCD, limbal tissue can be provided from a relative or a deceased individual, however, any non-autologous source requires prolonged immunosuppressive treatment.

To avoid the risks associated with prolonged use of immunosuppressants, numerous non-limbal autologous cell sources have been investigated for the treatment of bilateral LSCD in animal models over the past 13 years^4^. However, only cultured conjunctival epithelial cells^5^ and cultured oral mucosal epithelial cells (OMECs)^6^ have been evaluated in humans. Of these cell sources, OMECs are by far the most extensively studied^7^. However, the effects of the harvesting site and culture medium for generating an undifferentiated epithelium and sufficient cell growth have not yet been compared. Since 2010, following a study by Rama *et al*., the degree of differentiation of cultured epithelia for treating LSCD has been a major issue in corneal regenerative medicine^3^. Rama and colleagues demonstrated that transplantation of undifferentiated limbal epithelial sheets yields significantly better clinical results compared to the use of more differentiated equivalents. Thus, it may be clinically important to determine how the phenotype of cultured OMECs is influenced by the choice of tissue harvesting site and culture medium.

The culture media investigated in this study were chosen for the following reasons: EpiLife is serum-free and has a low calcium-content, which is known to promote an undifferentiated phenotype^8, 9^; oral keratinocyte medium (OKM) with oral keratinocyte growth supplement, which includes pituitary extract, protects cells from H_2_O_2_-induced cell death, DNA fragmentation, and has a positive effect on cell viability^10^; Dulbecco’s modified Eagle’s medium/Ham’s F12 (DMEM) and Roswell Park Memorial Institute 1640 medium (RPMI) are among the most commonly used cell culture media. Furthermore, cholera toxin and epidermal growth factors, which are typically added to DMEM, increase the proliferation capacity of oral epithelial cells^11^.

Although the harvesting site for culture of OMECs has not been described in all studies^12–17^, the most commonly reported harvesting site is the inferior buccal mucosa^6, 18–22^. However, no studies have compared various harvesting sites for *ex vivo* expanded OMECs. As the phenotype, degree of keratinization, and morphology of the oral mucosa vary throughout the oral cavity^23, 24^, we hypothesized that the harvesting site could affect the growth capacity and phenotype of *ex vivo* expanded OMECs.

In the current study, the effects of harvesting site and culture medium on attachment, growth, and phenotype of cultured OMECs were investigated. We found that OMECs from the lower lip and transition zone of the lower lip cultured in DMEM give rise to undifferentiated cells with high growth capacity, and hence are the most promising for treatment of LSCD.

## Methods

EpiLife medium, EpiLife defined growth supplement (EDGS), and trypsin-EDTA were purchased from Life Technologies (Grand Island, NY). Oral keratinocyte medium, oral keratinocyte growth supplement, and penicillin/streptomycin solution (P/S) were obtained from ScienCell Research Laboratory (Carlsbad, CA). Dulbecco’s modified Eagle’s medium/Ham’s F12, insulin, cholera toxin from vibro cholera, and human recombinant epidermal growth factor (EGF) were delivered by Sigma-Aldrich (St. Louis, MO). Roswell Park Memorial Institute medium 1640, 4-(2-hydroxyethyl)-1-piperazineethanesulfonic acid (HEPES), L-glutamine, non-essential amino acids (NEAA), and sodium pyruvate were obtained from Lonza (Walkersville, MD). Fetal bovine serum (FBS) was purchased from Hyclone Laboratories (Logan, UT). All cell culture and plastic wares were purchased from Thermo Fisher Scientific (Waltham, MA).

### Animal

Sprague-Dawley rats were used for the experiments. The Schepens Eye Research Institute (SERI) Animal Care and Use Committee approved the study employing rat oral mucosal tissue. All experiments using the animal were carried out in accordance with the approved guidelines.

### Explant Culture

**Figure 1:**
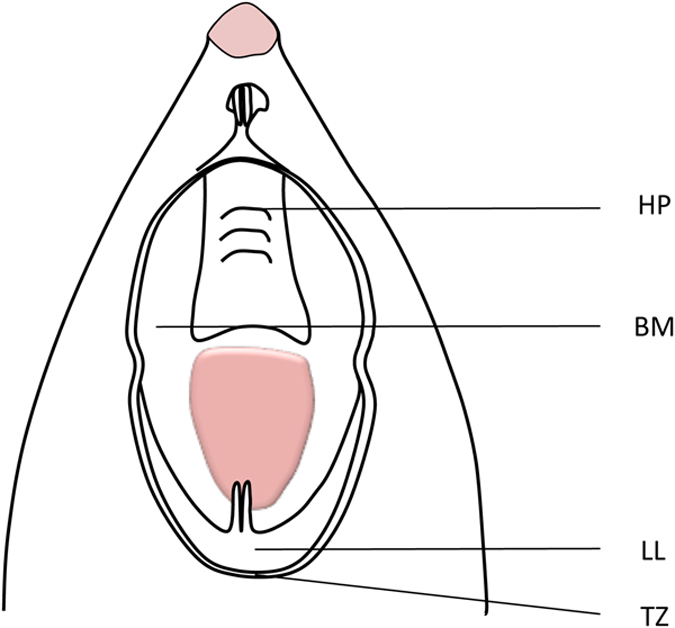
Schematic drawing of the rat oral cavity showing the oral mucosa tissue harvesting sites: hard palate (HP); buccal mucosa (BM); lower lip (LL); transition zone of the lower lip (TZ).

Oral mucosal epithelial cells were obtained from four harvesting sites: hard palate (HP), buccal mucosa (BM), lower lip (LL), and transition zone of the lower lip (TZ) of Sprague-Dawley rats (Fig. 1). The harvested tissue was rinsed three times with phosphate-buffered saline (PBS). The submucosal connective tissue was removed by dissection using forceps, scalpel, and a dissection microscope (Leica ZOOM 200, Leica Microsystems Inc., Buffalo, IL). The tissue samples were cut into 1–3 mm^2^ explants and immersed in the various media containing antibiotics (50 IU/ml P/S). The explants were transferred to 24-well tissue culture dishes, in which they were seeded with 180 *μ*l of culture medium to allow adhesion whilst still supplying nutrition. The explants attached to the surface within 24 to 48 hours. 500 *μ*l of culture medium was then added to each well to submerge the explants. The culture media used were: (1) EpiLife with EDGS, 50 IU/mL P/S (termed EpiLife in the present study); (2) OKM with oral keratinocyte growth supplement and 50 IU/mL P/S (termed OKM in the present study); (3) DMEM supplemented with 10% FBS, 5 *μ*g/mL insulin, 0.1 nM cholera toxin, 10 ng/mL human recombinant EGF, and 50 IU/mL P/S (termed DMEM in the present study); and (4) RPMI with 10% FBS, 2 mM L-glutamine, 87 *μ*M NEAA solution, 870 *μ*M sodium pyruvate, 8.7 mM HEPES, and 50 IU/mL P/S (termed RPMI in the present study). Explants were grown for either six or 13 days. Cultures were incubated at 37 °C with 5% CO_2_, and the medium was changed every other day.

### Cell Attachment and Outgrowth

For attachment analysis, explants from the four harvesting sites were cultured for 48 hours in culture wells containing 180 *μ*l of EpiLife, OKM, DMEM or RPMI. Explants were considered to be attached if after 48 hours, they were adherent to the culture well surface and did not float in the medium. The percentage of attached explants was calculated by dividing the total number of attached explants by the total number of seeded explants ×100%.

After six and 13 days in culture, the tissue outgrowth was measured for TZ and LL explants. To quantify the outgrowth, cultures were rinsed with PBS, fixed with 100% methanol for 30 minutes, and then rinsed again in PBS. Serial photographs were captured at 40x magnification using a phase contrast light microscope to visualize the entire outgrowth area of the culture. The outgrowth area was then quantified using ImageJ (National Institutes of Health, Bethesda, MD)^25^. Fold outgrowth was calculated using the following equation:

1 $$Fold\,out\,growth=\frac{Out\,growth\,area-explant\,area}{Explant\,area}$$

### Immunocytochemistry

Explants cultured for six days were fixed with methanol and incubated for one hour at room temperature in blocking buffer that consisted of 1% bovine serum albumin and 0.2% Triton X-100 dissolved in PBS. After the incubation, the cells were washed with fresh PBS and then incubated with primary antibodies overnight at 4 °C. Antibodies used in the study are listed in Table 1. Following incubation, the cells were washed three times with fresh PBS for 5 minutes on a shaker. Secondary antibodies conjugated to either Cy2 or Cy3 were used for the detection of protein-bound primary antibodies. The cells were incubated for 1 hour with secondary antibodies and then rinsed with PBS before mounting. A drop of photo-bleach protecting mounting media containing a 1:1000 dilution of 4′,6-diamidino-2-phenylindole (DAPI) was applied directly to the culture. A round glass coverslip was then placed on top of the culture before image acquisition. The cultures were visualized with an epifluorescence microscope (Olympus IX51; Center Valley, PA). Negative control consisted of replacing the primary antibodies with PBS. Expression of the markers was assessed by manual counting at 400x magnification. Cells were counted in photomicrographs captured near the explant and at the leading edge to assess if the degree of cell differentiation varied according to the distance from the explant^26^. Expression of proliferating cell nuclear antigen (PCNA) and nerve growth factor (NGF) p75 were calculated according to the following formula:

2 $$Percentage\,of\,positive\,cells=\frac{Number\,of\,positive\,cells}{Total\,number\,of\,cells}\times 100 \%$$

The remaining markers Bmi-1 (B lymphoma Mo-MLV insertion region 1 homolog), the transcription factor p63α, pan-cytokeratin (CK), and CK-4 were assessed using a semi-quantitative scoring system: cultures with less than 25% positive cells for the given marker were scored as ‘+’, between 25% and 50% were ‘++’, between 50% and 75% were ‘+++’ and more than 75% were ‘++++’. Cultures without positive cells were scored as ‘0’. Expression of all immunocytochemical markers was assessed by an experienced investigator blinded to the origin of the samples.

**Table 1:** List of Antibodies Used in the Study.

Antibody	Dilution
Anti-Bmi-1	1:100
Anti-p63α	1:100
Anti-pan-CK	1:100
Anti-CK-4	1:100
Anti-PCNA	1:200
Anti-NGF p75	1:400
Anti-Cy2	1:100
Anti-Cy3	1:300

Bmi-1 = B lymphoma Mo-MLV insertion region 1 homolog; CK = cytokeratin; PCNA = proliferating cell nuclear antigen; NGF = nerve growth factor; Cy = cyanine.

### Colony-Forming Efficiency Assay

Colony-forming efficiency (CFE) assay was performed to measure the growth capacity of cells from the LL, BM, HP, and TZ. Harvested tissue was exposed to 1.2 U/ml dispase diluted in the respective culture media for 1 hour to separate the epithelium from the basal membrane. The epithelial tissues were then incubated for five minutes in trypsin-EDTA (0.25%) at 37 °C to obtain a single cell suspension. The cells were seeded in six-well plates at a density of 2.5 × 10^3^/cm^2^. After 12 days of culture in DMEM or RPMI, the colonies were stained with crystal violet solution^27^. A colony was defined as a group of at least eight contiguous cells^28^. Colony-forming efficiency was calculated as follows:

3 $$CFE=\frac{Number\,of\,colonies}{Total\,number\,of\,cells\,seeded}\times 100 \%$$

The colonies stained with crystal violet solution were captured using a regular paper scanner. To assess the morphology of the cells light microscope was used.

### Statistical Analysis

The appropriate statistical test was determined with consideration of sample size and whether the data fitted a normal distribution or not. For comparisons of continuous variables of two groups, Mann-Whitney test was used, as the data was not always normally distributed. To compare three or more groups while adjusting for multiple testing, one-way analysis of variance (ANOVA) was used followed by a Tukey’s post-hoc test. *P* < 0.05 was considered significant. Values were presented as mean ± standard error of the mean (SEM).

## Results

### Effect of Culture Medium and Harvesting Site on Cell Attachment

**Figure 2:**
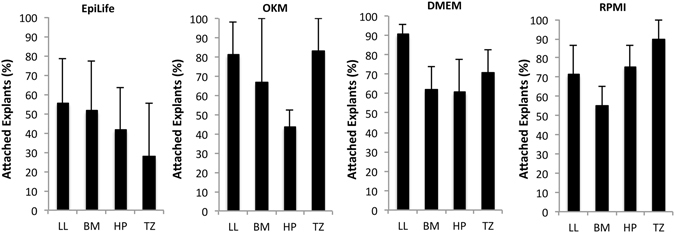
The percentage of attached explants following 48 hours of culture in four different media was compared using explants from four different rat oral mucosa harvesting sites. Each set of bar chart showing percentage of attached explants in EpiLife, oral keratinocyte medium (OKM), Dulbecco’s modified Eagle’s medium/Ham’s F12 (DMEM), and Roswell Park Memorial Institute 1640 medium (RPMI). The ability of the explants to attach to the culture wells was independent of both harvesting site and type of media.

Explant attachment to the culture substrate is a pre-requisite for epithelial cell outgrowth. The percentage of attached explants was compared between each culture medium and harvesting site used. Location wise, the percentages of attached explants in EpiLife were 55%, 51% 41%, and 27% for LL, BM, HP and TZ, respectively. For OKM the attachment percentages were 81%, 66%, 43%, and 83% for LL, BM, HP and TZ, respectively. In DMEM the percentages were 90%, 62%, 60% and 70% for LL, BM, HP and TZ, respectively. In RPMI the percentage values were 71%, 55%, 75% and 90% for explants harvested from LL, BM, HP and TZ respectively (Fig. 2). Neither choice of media or harvesting site revealed any statistically significant difference in the ability of the explants to attach to the culture well.

### Effect of Culture Medium and Harvesting Site on Cell Outgrowth

**Figure 3:**
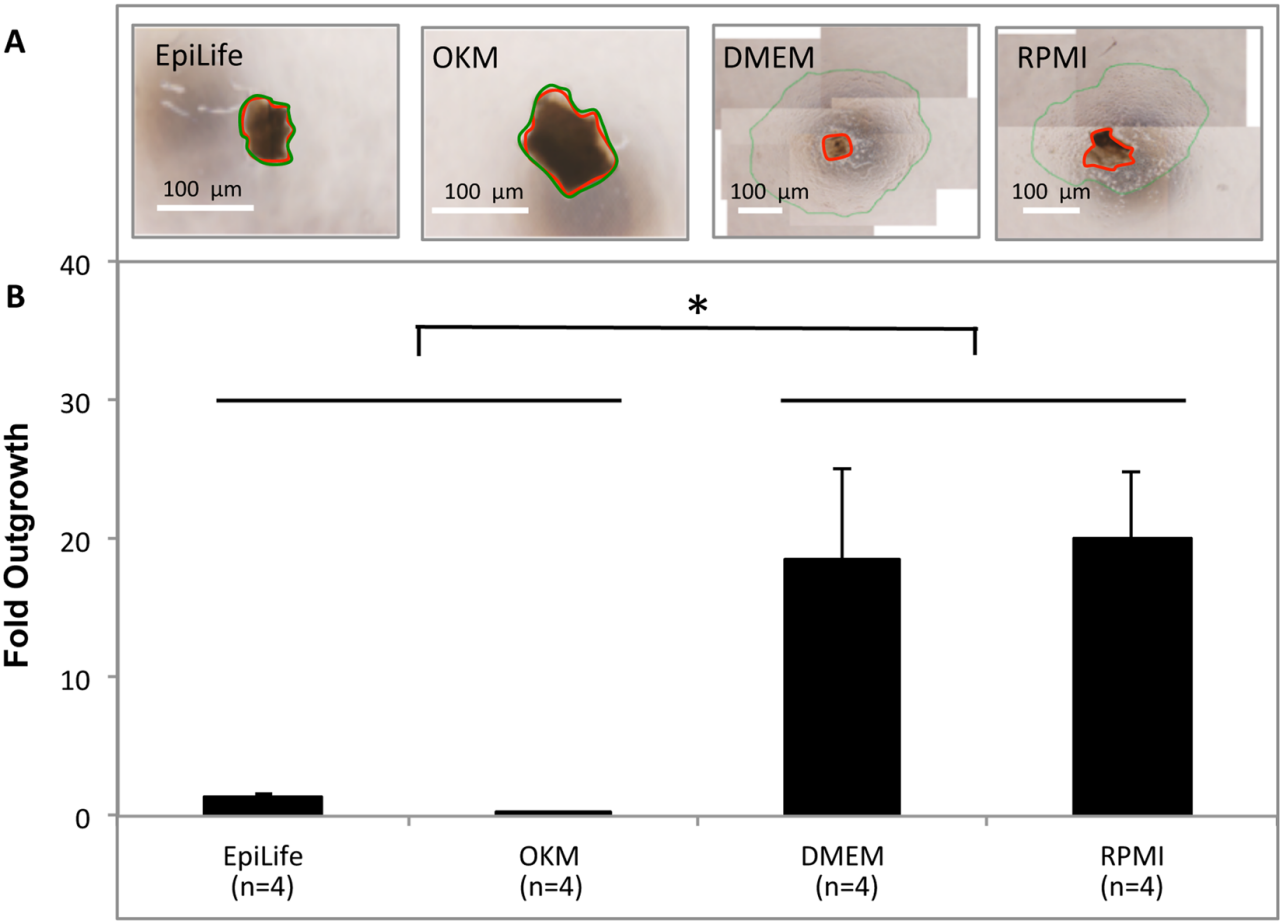
(**A**) Light microscopy images showing representative outgrowth of epithelial cells from rat oral mucosa explants from lower lip region cultured for six days in EpiLife, oral keratinocyte medium (OKM), Dulbecco’s modified Eagle’s medium/Ham’s F12 (DMEM), and Roswell Park Memorial Institute 1640 medium (RPMI). Red circles indicate the explant area. Green circles indicate the leading edge of the cellular outgrowth. The area between the red and the green circle represents the outgrowth area. (**B**) Bar chart showing fold outgrowth in EpiLife, OKM, DMEM, and RPMI. *P < 0.05. Fold outgrowth was calculated by dividing the outgrowth area by the explant area. Images are representative of four experiments.

Fold outgrowth in relation to explant size was calculated following six-day culture using the four culture media (Fig. 3A). EpiLife (1.4 ± 0.2) and OKM (0.04 ± 0.03) gave rise to a low fold outgrowth, which was lower than that promoted by DMEM (18 ± 6; *P* = 0.044 and *P* = 0.027, respectively) and RPMI (20 ± 5; *P* = 0.03 and *P* = 0.018, respectively) (Fig. 3B). Therefore, all subsequent experiments were performed using only DMEM or RPMI.

**Figure 4:**
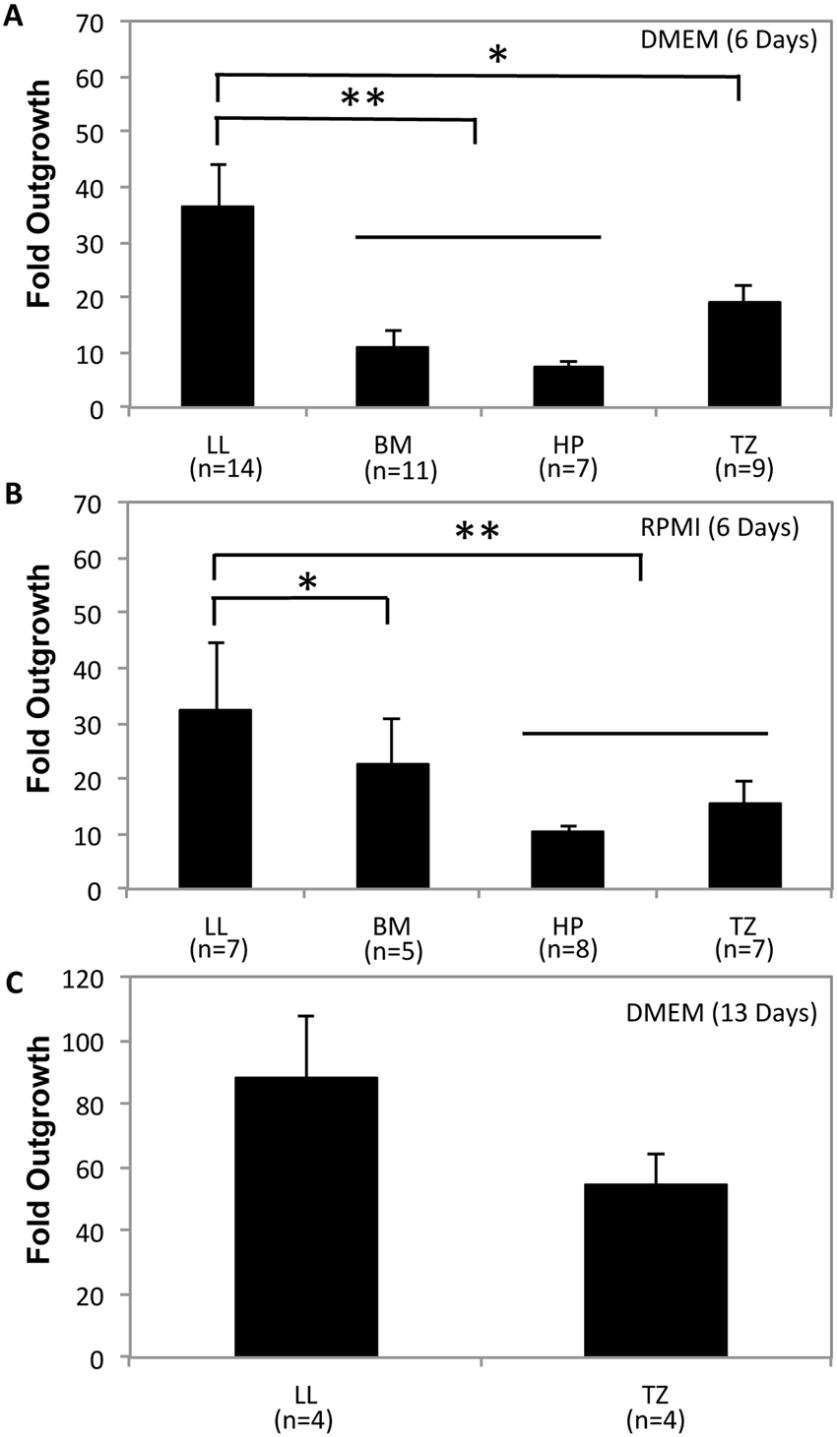
Bar charts showing fold outgrowth from rat oral mucosa explants harvested from the lower lip (LL), buccal mucosa (BM), hard palate (HP), and transition zone of the lower lip (TZ). (**A**) Explants were cultured in Dulbecco’s modified Eagle’s medium/Ham’s F12 (DMEM) or (**B**) Roswell Park Memorial Institute 1640 medium (RPMI) for six days. (**C**) Fold outgrowth from LL and TZ explants was also measured after 13 days of culture in DMEM. *P < 0.05 and **P < 0.005.

We then assessed fold outgrowth following six-day culture using explants from the four harvesting sites. When cultured in DMEM, explants from LL (36 ± 7) generated a higher fold outgrowth than explants from BM (11 ± 3; *P* < 0.001), HP (7 ± 0.7; *P* < 0.001), and TZ (19 ± 3; *P* = 0.046) (Fig. 4A). Similarly, explants from LL cultured for six days in RPMI (32 ± 12) generated significantly higher fold outgrowth than explants from BM (22 ± 8; *P* = 0.044), HP (10 ± 1; *P* < 0.001), and TZ (15 ± 4; *P* = 0.005) (Fig. 4B).

Fold outgrowth was also measured after long-term culture in DMEM. After 13 days of culture, the fold outgrowth from LL explants did not differ significantly from that of TZ explants (Fig. 4C). Collectively, our results show that DMEM and RPMI yielded the highest fold outgrowth, whereas explants from LL gave the biggest fold outgrowth following six-day culture and comparable outgrowth to TZ following 13-day culture.

### Effect of Culture Medium and Harvesting Site on Cell Phenotype

To determine the effect of the choice of culture medium, as well as tissue harvesting site, on phenotype of cells in primary culture, the expression of immunocytochemical markers related to differentiated and undifferentiated cells (Table 1) was compared among the groups.

**Figure 5:**
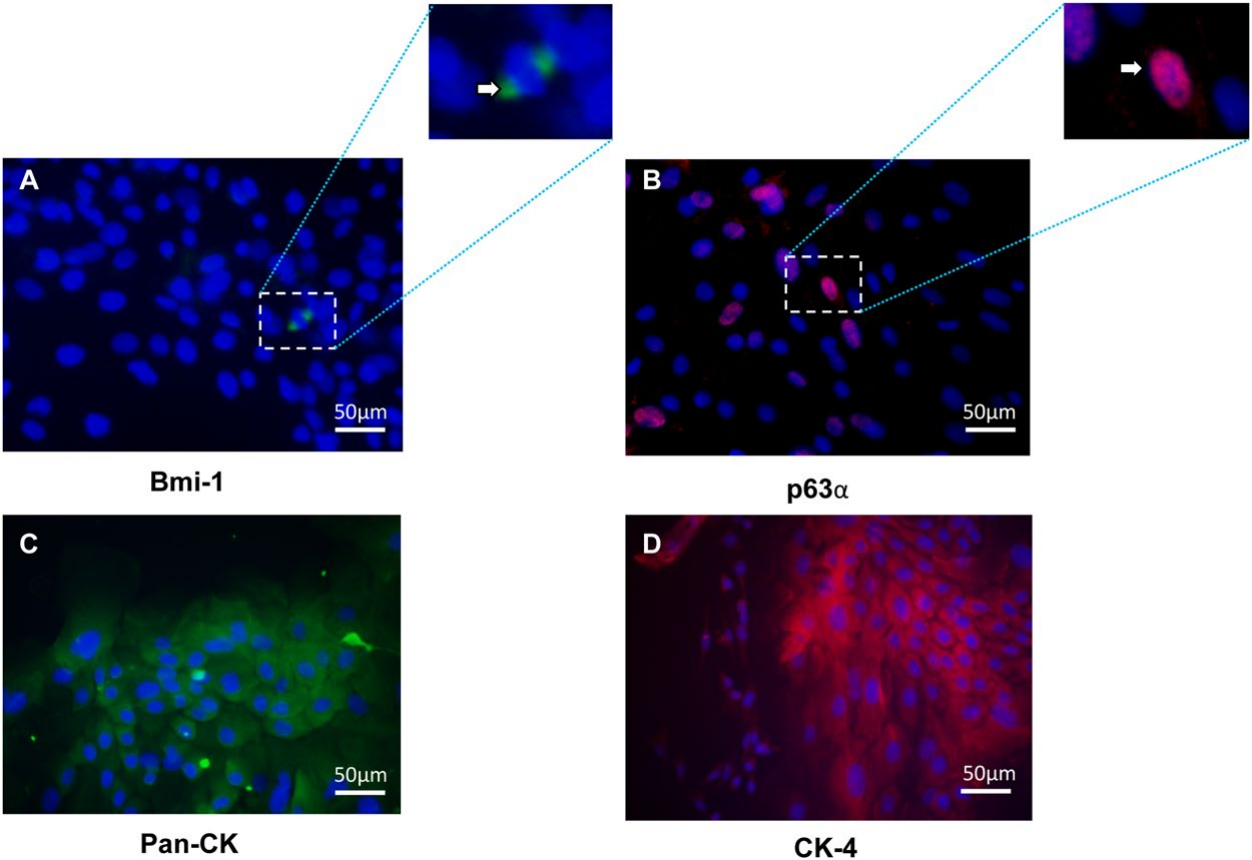
Photomicrographs showing immunostaining of the putative stem cell markers Bmi-1 (green) (**A**) and p63α (red) (**B**), as well as the epithelial cell marker anti-pan-cytokeratin (CK; green) (**C**) and the stratified squamous epithelial cell marker anti-CK-4 (red) (**D**), in rat oral mucosal epithelial cells from lower lip cultured for six days in Dulbecco’s modified Eagle’s medium/Ham’s F12. Images are representative of three samples. Nuclei were counterstained with DAPI (blue). The arrow in image A indicates Bmi-1 staining (green). The arrow in image B indicates p63α staining (red). Magnification: 400x.

We first investigated the expression of undifferentiated cell markers Bmi-1^29^ and the transcription factor p63α^30^. Bmi-1, a protein required for maintenance of adult stem cells^31^, was detected in the cell nucleus of only a few cells (Fig. 5A). p63α was detected in a minority of cell nuclei (Fig. 5B). Most of the cultured cells demonstrated cytoplasmic staining for the epithelial cell marker pan-CK (Fig. 5C) and the stratified squamous epithelial cell marker CK-4 (Fig. 5D).

Bmi-1 positive cells were detected in fewer than 25% of the cells from all harvesting sites cultured in DMEM. However, in cultures from HP and TZ, Bmi-1 was undetectable at the leading edge (Table 2). In DMEM, no p63α positive cells were observed at the leading edge of the cultures originating from LL and HP, whereas p63α positive cells were detectable in fewer than 25% of the cells at the leading edge of BM and TZ cultures. Near the explant, fewer than 25% of the cells were positive for p63α in cultures from LL, BM, and HP, whereas in cultures from TZ 25% to 50% of the cells near the explant were p63α positive. With the exception of BM cultures, cultures from all other harvesting sites showed a lower percentage of p63 positive cells at the leading edge than near the explant (Table 2). In DMEM, the percentages of both pan-CK and CK-4 positive cells were similar (75–100%) irrespective of harvesting site and irrespective of the location of the expanded cells (i.e., near the explant or at the leading edge), indicating that the absolute majority of the cultured cells were epithelial (Table 2).

**Table 2:** Immunocytochemical Staining of Rat Oral Mucosal Epithelial Cells Cultured in DMEM.

Antibody	Tissue Harvesting Site							
	LL		BM		HP		TZ	
	Near explant	At leading edge	Near explant	At leading edge	Near explant	At leading edge	Near explant	At leading edge
Anti-Bmi-1	+	+	+	+	+	0	+	0
Anti-p63α	+	0	+	+	+	0	++	+
Anti-pan-CK	++++	++++	++++	++++	++++	++++	++++	++++
Anti-CK-4	++++	++++	++++	++++	++++	++++	++++	++++

Immunocytochemical staining was scored as 0 (undetectable), +(detectable in < ¼ of the cells), ++(detectable in ¼–½ of the cells), +++ (detectable in ½–¾ of the cells), and ++++ (detectable in >¾ of the cells). N = 3–5. Scores were assigned by an experienced investigator blinded to the origin of the samples. DMEM = Dulbecco’s modified Eagle’s medium/Ham’s F12; LL = lower lip; BM = buccal mucosa; HP = hard palate; TZ = transition zone of the lower lip; Bmi-1 = B lymphoma Mo-MLV insertion region 1 homolog; CK = cytokeratin.

In contrast to DMEM, none of the cultures grown in RPMI displayed Bmi-1 positive cells (Table 3). In RPMI, up to 50% of the cells were positive for p63α irrespective of both the location in culture and of the harvesting site. Only cultures derived from LL and TZ showed a lower percentage of p63α positive cells at the leading edge (<25%) compared to near the explant (25–50%) (Table 3). As for cultures grown in DMEM, a similar percentage of pan-CK and CK-4 positive cells were obtained from all harvesting sites when growing cells in RPMI (Table 3).

**Table 3:** Immunocytochemical Staining of Rat Oral Mucosal Epithelial Cells Cultured in RPMI.

Antibody	Tissue Harvesting Site							
	LL		BM		HP		TZ	
	Near explant	At leading edge	Near explant	At leading edge	Near explant	At leading edge	Near explant	At leading edge
Anti-Bmi-1	0	0	0	0	0	0	0	0
Anti-p63α	++	+	+	+	+	+	++	+
Anti-pan-CK	++++	++++	++++	++++	++++	++++	++++	++++
Anti-CK-4	++++	++++	++++	++++	++++	++++	++++	++++

Immunocytochemical staining was scored as 0 (undetectable), +(detectable in <¼ of the cells), ++ (detectable in ¼–½ of the cells), +++ (detectable in ½–¾ of the cells), and ++++ (detectable in >¾ of the cells). N = 3–5. Scores were assigned by an experienced investigator blinded to the origin of the samples. RPMI = Roswell Park Memorial Institute 1640 medium; LL = lower lip; BM = buccal mucosa; HP = hard palate; TZ = transition zone of the lower lip; Bmi-1 = B lymphoma Mo-MLV insertion region 1 homolog; CK = cytokeratin.

**Figure 6:**
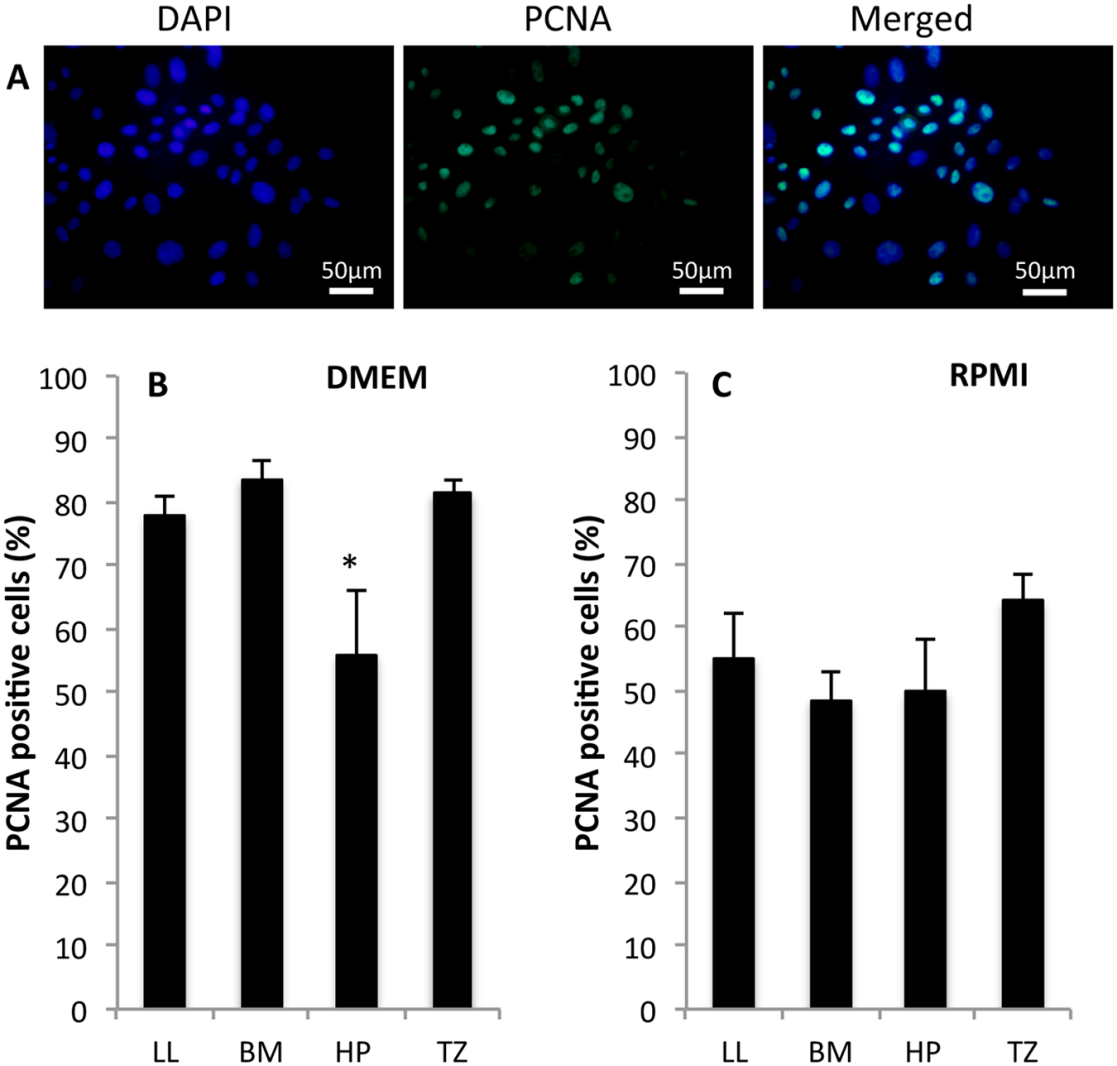
Rat oral mucosal epithelial cells were cultured for six days and then immunostained with anti-proliferating cell nuclear antigen (PCNA) to assess the percentage of proliferating cells. (**A**) Corresponding photomicrograph of cultured cell nuclei stained with DAPI (blue; left and right image) and immunostained with anti-PCNA (green; middle and right image). The cells were harvested from the lower lip (LL) oral mucosa and cultured in Dulbecco’s modified Eagle’s medium/Ham’s F12 (DMEM). Images are representative of three samples. Magnification: 400x. (**B**) PCNA expression of cultured cells in DMEM from explants harvested from LL, buccal mucosa (BM), hard palate (HP), and transition zone of the lower lip (TZ) (**C**) PCNA expression of cultured cells in RPMI from explants harvested from LL, BM, HP, and TZ. *P < 0.05.

The percentage of proliferative cells was assessed by counting PCNA positive cells following six-day culture in DMEM or RPMI. Positive staining for PCNA was seen in cell nuclei (Fig. 6A). PCNA positive cells cultured in DMEM were significantly lower in culture from HP compared to the culture from LL (*P* = 0.012), BM (*P* = 0.014) and TZ (*P* = 0.009) (Fig. 6B). Explants cultured in RPMI harvested from LL, BM, HP, and TZ, showed comparable percentages of PCNA positive cells in all cultures (Fig. 6C).

**Figure 7:**
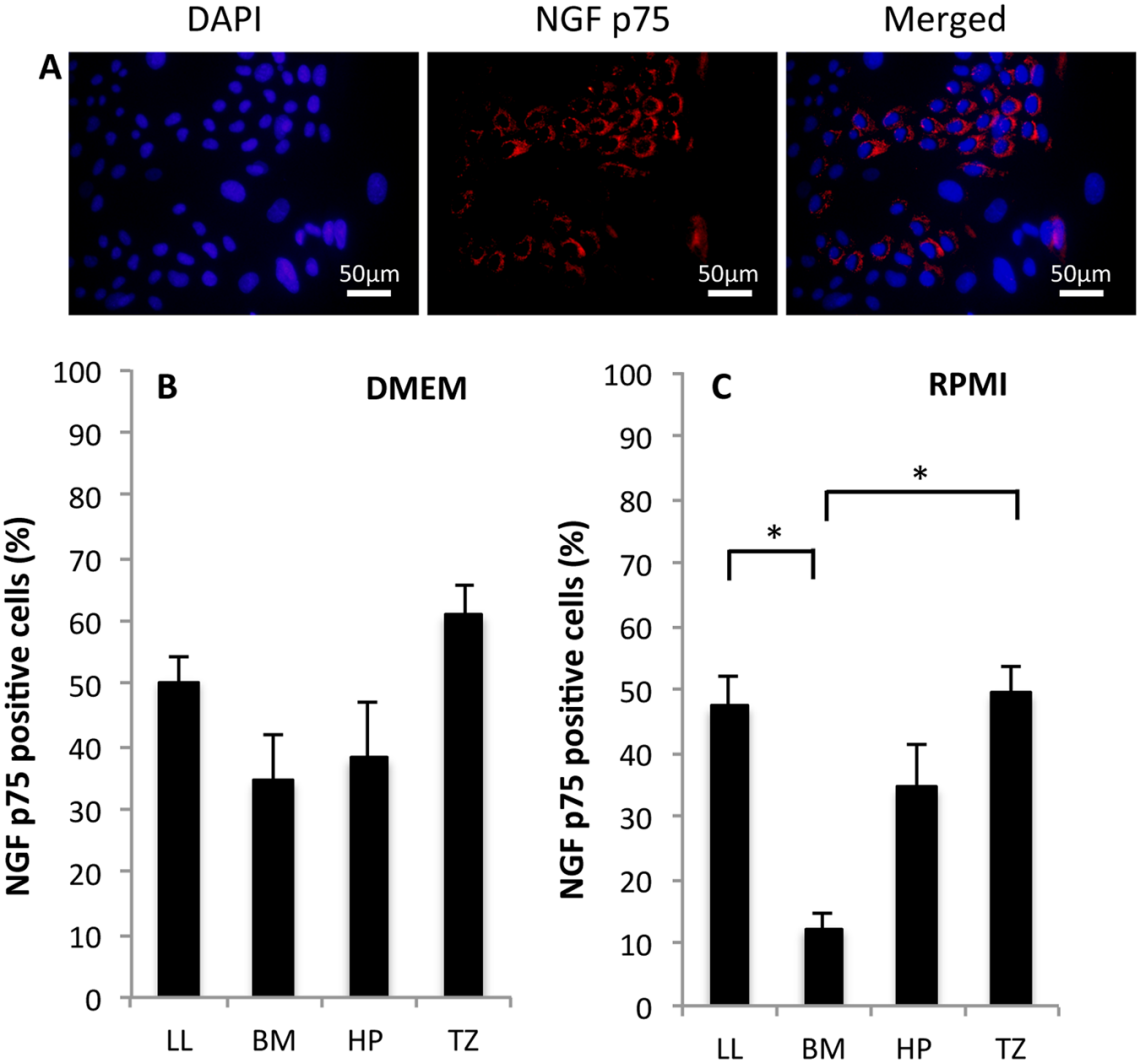
Rat oral mucosal epithelial cells were cultured for six days and then immunostained with anti-nerve growth factor (NGF) p75 to assess the percentage of undifferentiated cells. (**A**) Corresponding photomicrograph of cultured cell nuclei stained with DAPI (blue; left and right image) and immunostained with anti-NGF p75 (red; middle and right image). The cells were harvested from the lower lip (LL) oral mucosa and cultured in Dulbecco’s modified Eagle’s medium/Ham’s F12 (DMEM). Images are representative of three samples. Magnification: 400x. (**B**) NGF p75 expression of cultured cells in DMEM from explants harvested from LL, buccal mucosa (BM), hard palate (HP), and transition zone of the lower lip (TZ) (**C**) NGF p75 expression of cultured cells in RPMI from explants harvested from LL, BM, HP, and TZ. *P < 0.001.

The undifferentiated cell marker NGF p75 was detected in the cytoplasm of the cultured cells (Fig. 7A). The percentage of cells positive for NGF p75 in DMEM cultures derived from LL, BM, HP, and TZ were 50% ± 4%, 34 ± 8%, 38 ± 9%, and 61% ± 5%, respectively (Fig. 7B). Culture derived from LL, BM, HP, and TZ in RPMI, showed 47% ± 5%, 12% ± 2%, 34% ± 7%, and 49% ± 4% percentage of NGF p75 positive cells, respectively. BM cultures had a significantly lower percentage of NGF p75 positive cells compared to the cultures derived from LL (*P* < 0.001) and TZ (*P* < 0.001) (Fig. 7C). Together, these results suggest that an undifferentiated phenotype is supported in both DMEM and RPMI. However, expression of NGF p75 varies depending on the harvesting location in DMEM. Furthermore, explants from LL and TZ tended to support a higher percentage of proliferative cells and a more undifferentiated phenotype than explants from HP and BM, respectively.

### Effect of Culture Medium and Harvesting Site on Colony-Forming Efficiency

**Figure 8:**
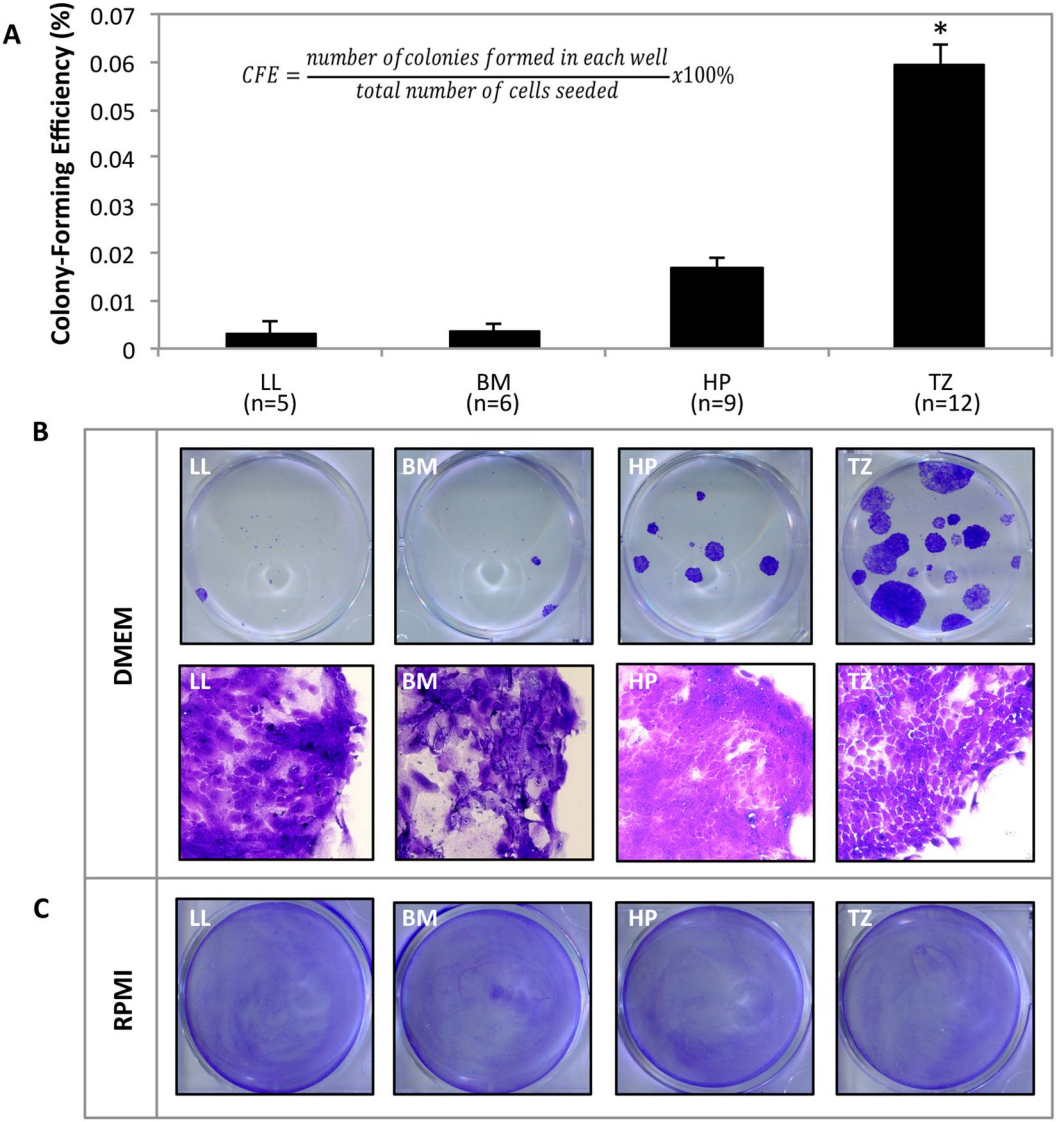
Rat oral mucosal epithelial cells harvested from four sites in the oral cavity (lower lip (LL), buccal mucosa (BM), hard palate (HP), and transition zone of the lower lip (TZ)) were assayed for colony-forming efficiency (CFE) for 12 days in Dulbecco’s modified Eagle’s medium/Ham’s F12 (DMEM) or Roswell Park Memorial Institute 1640 medium (RPMI). (**A**) Bar chart showing the percentage of colonies formed when using DMEM as culture medium. *P < 0.001. (**B**) Photographs (top row) show representative colonies grown in DMEM and stained with crystal violet. Photomicrographs (bottom row; magnification: 200x) show corresponding bright-field images of the clonal cells stained with crystal violet. Images are representative of at least five samples. (**C**) Photographs showing no colonies when cells were cultured in RPMI. Images are representative of three samples.

A colony-forming efficiency assay was performed to assess clonal growth capacity. In DMEM, LL (0.003% ± 0.002%), BM (0.003% ± 0.001%), and HP (0.016% ± 0.002%) cells yielded lower CFE than TZ (0.593% ± 0.004%; *P* < 0.001) (Fig. 8A). Cells from LL and BM gave rise to the smallest colonies (presumed paraclones), cells from HP yielded medium-sized colonies (presumed meroclones), whereas cells from TZ gave rise to the largest colonies (presumed holoclones) (Fig. 8B). In contrast to DMEM, RPMI did not give rise to any colonies after 12 days of culture (Fig. 8C).

## Discussion

In the present study, OMECs were harvested from four sites in the oral cavity of rats and cultured in four different media (EpiLife, OKM, DMEM, and RPMI) and the explant attachment, cell outgrowth, proliferation, and phenotype of the cells were compared. Our results indicate that OMECs cultured in EpiLife, OKM, DMEM, and RPMI have similar attachment ability. Epithelial cells are anchorage dependent, and therefore cell adhesion is necessary for their proliferation^32^.

DMEM and RPMI yielded a higher fold outgrowth of cells than EpiLife and OKM. The presence of growth promoting supplements in the media plays an important role in *ex vivo* cell proliferation^33–35^. Formanek *et al*. found that among numerous tested additives, DMEM supplemented with fetal calf serum, EGF, insulin, and hydrocortisone exhibited the greatest capacity for promoting oral keratinocyte growth^36^. Our finding that DMEM generated a higher fold outgrowth than OKM and EpiLife is consistent with this study^36^. However, RPMI yielded similar fold outgrowth despite not being supplemented with EGF, insulin, and hydrocortisone.

In the current study, we were able to produce undifferentiated epithelial sheets in several of our cultures. An undifferentiated phenotype in cultured limbal epithelial stem cell transplants has been found to be a strong predictor of clinical success following transplantation in patients with LSCD^3^. Further studies are warranted to explore the effects of the phenotype of OMCEs on clinical results following transplantation.

Kolli *et al.* reported a loss of p63 positive cells with increasing distance from limbal explants^37^. In agreement with this study, we found a lower percentage of p63α positive cells at the leading edge than near the explant in cultures from LL and TZ grown in DMEM or RPMI^26^. Cells harvested from HP cultured in DMEM, but not in RPMI, also showed a lower percentage of p63α positive cells at the leading edge than near the explant. Bmi-1, another putative stem cell marker applied in our study, demonstrated a similar tendency, strengthening the argument that increasing distance from the explant results in a higher degree of differentiation of cultured epithelial cells. As an undifferentiated phenotype is considered highly advantageous in corneal regenerative medicine^3^, our study also lends support to the idea of using shredded explants for treating LSCD^38^, especially if the explants are spread apart in the culture wells. Considering our observations together with the reports from Kolli *et al*., two cell types appear to change phenotypically with respect to the distance from the explant. Further studies are warranted to explore whether this tendency appears to be irrespective of cell types and, thus, represents a general principle in *ex vivo* expansion of cells from explants for use in regenerative medicine^4^.

Pan-CK and CK-4 expression were detected in more than 75% of the cells across both DMEM and RPMI. This finding indicates that the majority of the cultured cells were epithelial cells.

Cultures from HP had a significantly lower expression of the proliferation marker PCNA than cultures from LL, BM and TZ, which agrees with our finding that explants from HP demonstrated the least outgrowth.

Nerve growth factor p75 has been suggested as a stem/progenitor cell marker for OMECs and cells positive for this marker typically cluster in specific regions of the oral mucosal epithelium^39^. It has been reported that NGF p75 positive cells have a high *in vitro* proliferative capacity and clonal growth potential^39^. In RPMI, explants from BM gave rise to the lowest percentage of NGF p75 positive cells.

Buccal mucosa has been employed as harvesting site for OMEC transplants in several studies^6, 12, 13, 16–21, 40–48^. However, our study showed that OMECs from TZ yielded higher CFE than BM. RPMI did not yield any colonies nor did the cells cultured in this medium express Bmi-1. The ability of OMECs to form colonies may be related to the maintenance of Bmi-1 expression as Bmi-1 was found to be related to the self-renewal capacity of human limbal epithelial stem cells^49^. We did not use a feeder layer when performing the CFE assay in our study. In the absence of a feeder layer, a cyclic adenosine monophosphate inducer such as cholera toxin, has been shown to be necessary for rat OMECs to form colonies^50^. This is in agreement with our results in which we only obtained colonies when culturing the cells in a cholera toxin-supplemented medium (DMEM).

Explants from LL and TZ exhibited a higher proliferative capacity than explants from BM and HP, as demonstrated by measurement of fold outgrowth and CFE. Stem cells reside in a particular microenvironment known as a niche^51^. One benefit of using explants for *ex vivo* expansion of OMECs is that the stem cells remain in, and are supported by, their niche, which is known to control stem cell function^52^. Asaka *et al*. showed that label-retaining cells in rat oral mucosal epithelium, which may represent stem cells, were localized to the basal layer. Where the epithelium had rete ridges the label-retaining cells were seen at the bottom of these ridges, however, where the epithelium had less pronounced rete ridges the label-retaining cells were randomly located in the basal layer^53^. In our study, we harvested tissues from the BM and LL (representing lining mucosa), where epithelial rete ridges are shallow and gentle^39, 54^, and HP (representing masticatory mucosa), where they are steep and deep^39, 54^. No study has directly compared the rete ridges of rats in LL, BM, HP, and TZ. However, TZ in rodents has been shown to have more prominent rete ridges than the adjacent epidermis^55^. The higher percentage of colonies formed from TZ cells compared to the other three harvesting sites tested in our study infers a relatively larger stem cell content in TZ. As TZ has prominent rete ridges this increases the surface area of the basement membrane and therefore the relative number of basal cells (where stem cells are likely to be located) compared to supra-basal cells. Analogous to this, thin epithelia have been shown to have higher CFE than thick epithelia, possibly due to a relatively larger content of basal stem cells^50^. Compared to cells from the other three tested harvesting sites, TZ cells could also be less dependent on their niche for colony-formation. However, as LL explants yielded higher fold outgrowth than TZ, we speculate that: (1) LL has a relatively higher percentage of transient amplifying cells with high proliferative capacity; (2) outgrowth of LL cells is more dependent on the stem cell niche (which is included with explant culture) than TZ cells; or (3) outgrowth of LL cells is dependent on a higher cell density (which is achieved when using explants rather than cell suspension) than TZ cells. Cell density has been shown to greatly affect proliferation of rat OMECs^50^. An in-depth analysis of differences in the stem cell niches of the four harvesting sites is needed to provide a more definite answer.

In conclusion, the choice of tissue harvesting site and culture medium influence the attachment, growth, and phenotype of cultured OMECs. Explants from LL and TZ cultured in DMEM are more favorable than BM. This is particularly interesting as BM has been the by far most commonly reported harvesting site to generate OMEC sheets for transplantation. Comparative studies are warranted to assess whether optimization of the choices of harvesting site and culture medium can improve the clinical success of transplantation of cultured OMEC to patients with LSCD.
